# Design and Experimental Validation of a Switchable Frequency Selective Surface with Incorporated Control Network

**DOI:** 10.3390/s23094561

**Published:** 2023-05-08

**Authors:** Andrei-Marius Silaghi, Farzad Mir, Aldo De Sabata, Ladislau Matekovits

**Affiliations:** 1 Department of Measurements and Optical Electronics, Politehnica University Timisoara, 300006 Timisoara, Romaniaaldo.de-sabata@upt.ro (A.D.S.); 2 Department of Electrical and Computer Engineering, University of Houston, Houston, TX 77204, USA; farzadmir1992@gmail.com; 3 Department of Electronics and Telecommunications, Politecnico di Torino, 10129 Turin, Italy; 4 Istituto di Elettronica e di Ingegneria dell’Informazione e delle Telecomunicazioni, National Research Council, 10129 Turin, Italy

**Keywords:** frequency selective surface, switchable, cut-slot, control network

## Abstract

Tunable/switchable devices are more and more required in modern communication systems. However, the realization of the tuning requires the presence of active devices, which in turn must be biased. The current paper comes up with a solution for designing and experimentally validating such a switchable Frequency Selective Surface. Two different metallic structures are simulated and measured, having incorporated the same topology control network (CN). In this scenario, the main innovation of this paper is the presence of the feeding part, namely the control network. In this work, the main FSS structure is flanked by three parallel CN microstrip lines and several via holes that allow biasing the active elements, namely PIN diodes. The switchability of the proposed structure is achieved through PIN diodes, whose bias determines the values of the elements in the equivalent circuit. At different biases, the response of the FSS changes accordingly. From all possible values of the bias, the extreme cases when the diodes act as (almost) short- and open-circuits are considered in the submitted manuscript for the sake of brevity. These cases are modeled by the main and cut-slot structures, respectively. The proposed structures have been evaluated using electromagnetic simulation and implemented on an FR4 substrate having a thickness of 1.58 mm. With the periodicity of the square-shaped unit cell of 18 mm edge length, different filtering bands are obtained below 12 GHz. Another novelty that has received very little consideration in the existing literature is the use of a finite array of unit cells instead of an infinite one. And finally, tests in an anechoic chamber have proved that there is a good agreement between practical and simulation results and also demonstrated the proper performance of the devices for wide angular incidence for both TE and TM polarizations.

## 1. Introduction

Real-time control of the dispersion properties of single unit cells of a periodic structure [1] allows for a quick change in the frequency response of the device incorporating it. Such opportunities find application in various fields of science, extending from acoustics to electromagnetics, from optics to mechanics, etc. Propagation of electromagnetic waves depends on the media parameters, which in turn can be used to tailor phase front and amplitude, i.e., signal strength, in any given direction of interest [1].

Metasurfaces (MSs), with sub-wavelength dimensions of unit cells and thickness, represent an elegant and space-saving solution to do so, aiming to achieve desired beam forming, control of direction of propagation, frequency-dependent intensity, phase, polarization state, etc. For example, in modern 5G communication systems, controllable MSs are proposed to be integrated in the structure of the antenna arrays to facilitate tracking by the base station of fast mobile users as a cost-effective solution [2].

Frequency selective surfaces (FSSs) are also 2D periodic structures with applications such as spatial filtering, screening, and polarization conversion when linearly polarized incident waves are converted into circular polarized ones, which are less sensitive to distortions introduced by harsh propagation environs, e.g., human body etc. refs. [3,4,5]. A natural evolution of such structures consists of making them adaptive, i.e., changing their response according to different transmission/reflection requirements. Tunable/programmable FSSs have promising potential in other areas as well, e.g., optical and spatial light modulators [6], holography [7], acoustics [8,9].

The primary drawback of typical FSS structures is their dependence on the angle of incidence of electromagnetic waves for their reflection and transmission properties. As solutions to this problem and in order to design tunable/switchable FSSs, engineers are increasingly incorporating lumped elements into designs, using two main variants: by means of varactor diodes or PIN diodes [10,11].

Furthermore, a DC bias network (Control network-CN) must be utilized in designs with active components. Microstrip lines implanted in the substrate can be thought of as feeding lines for biasing the nonlinear elements rather than biasing them directly inside the structure through several wires [11,12]. The topic of designing tunable/switchable FSSs devices, including FSSs, is one of the most challenging in many fields of science, as demonstrated by the large number of research groups wordwide working on it [9,10,11,12,13,14,15,16].

The implementation of the dynamic response is the most challenging part of the proposed design. In the recent literature, a variety of solutions exist that make use of different kinds of excitations to control the propagation of the carrier signal: photo-responsive elastic metamaterials or thermal tunability are just some of the techniques used to control the propagation channel [17,18].

The design of a switchable FSS is presented in this paper. To overcome the previously mentioned issues, a microstrip-line-based CN is proposed; it is located at the bottom of the composition, so no additional space is required to accommodate it. Via-holes are implemented to ensure the connection between the CN and the principal structure. In the present study, the ON and OFF states of the diodes are ideally considered (modeled as short circuit and open, respectively). These two configurations have been analyzed and experimentally characterized separately. Both configurations incorporate the same CN geometry.

The multiple stop-band configurations are implemented on a single-layered, double-face, cost-effective FR4 substrate. The unit cell extends over 18 × 18 mm${}^{2}$. Experiments using a board made up of 12 × 12 unit cells were successfully used to validate numerical simulations.

The rest of the article is structured as follows. The proposed solutions are discussed in Section 2, after which an electromagnetic simulation is used to evaluate the operation. Field images for the structures and CN lines are generated and explained in order to give more depth to the work. Furthermore, parametric variations are tackled in this section to demonstrate the switchability of the structures. In Section 3, the effect of using a finite array of unit cells rather than an infinite one is assessed. Arrays of 6 × 6 and 8 × 8 unit cells have been investigated due to the fact that the practical validation uses more than one unit cell on a board. Experimental results are reported in Section 4, practical tests are made in an anechoic chamber, and conclusions are drawn in the last section.

## 2. Design of Unit Cells

In this section, the design of both a main structure and a cut-slot structure with a symmetric unit-cell is presented. However, it is to be noted that the presence of the CN will break the initial symmetry.

In the present design, cuts to separate different resonators with diverse resonant frequencies have been used, namely the inner, central, and outer rings. In a future design, each of these cuts will be “filled” with a PIN diode. Here, four cuts are considered in the unit cells between the inner and central rings, and similarly, four cuts are considered for the separation of the central resonator from the external one. Since the structure’s frequency response depends on the geometry, the insertion of such cuts will modify the internal resonances of the geometry. In an ideal case, the equivalent circuit of a diode corresponds to “short circuit” (when the diode is in conduction) and “open circuit” (when the diode is in an “off” state). These two cases have been considered in separate manifestations and named the “main structure” and “cut-slot”, respectively. Like previously stated, the authors chose to use two designs, namely a main structure and a cut-slot one, in order to simulate the behavior of the structures in the presence of diodes (ON state-main structure and OFF state-cut-slot structure).

**Figure 1:**
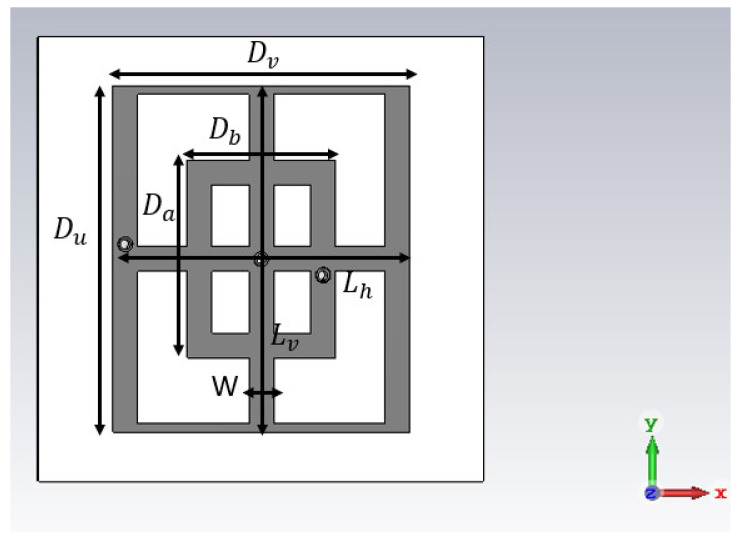
Top view of the unit cell of the main structure.

**Figure 2:**
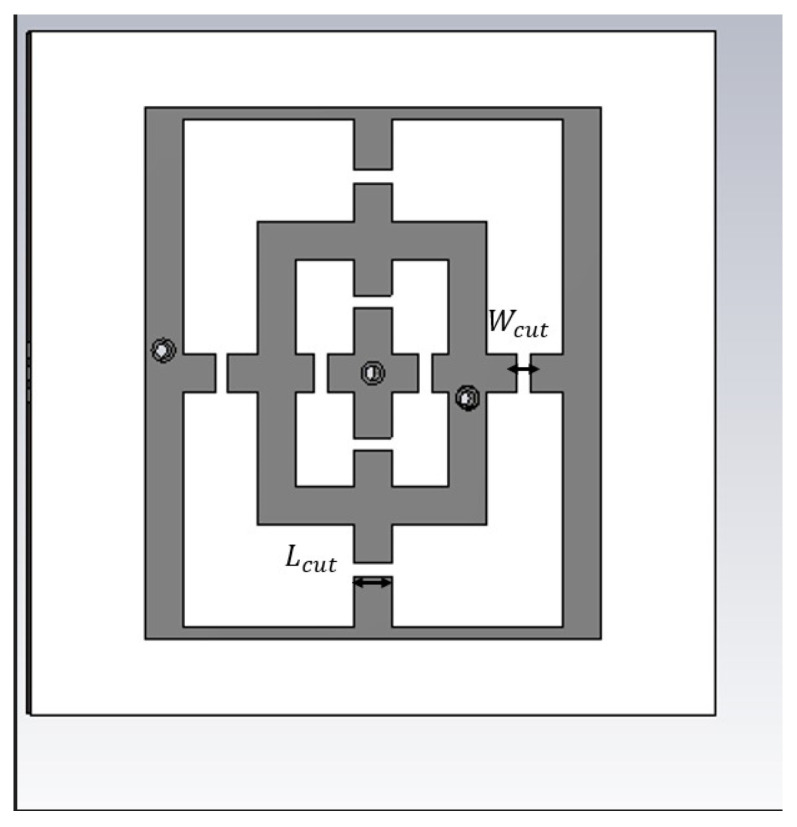
Top view of the unit cell of the cut-slot structure.

Figure 1 and Figure 2 illustrate the structures of the unit-cell (top view) that incorporate metallic rectangular-shaped loadings built on the FR4 substrate layer, with $\epsilon_{r}=3.9$ and tanδ=0.025. The metallic regions are depicted with grey color (dark). The difference between the two plots, consisting of eight cut-slots is explained below. The thickness of the structures is $h_{1}=1.58$ mm. The dimensions of the structures are defined in Figure 1 as follows: the large rectangle sides are $D_{u}=14$ mm, $D_{v}=12$ mm, while for the small one the sides are $D_{a}=8$ mm, $D_{b}=6$ mm. These two rectangles are connected through two cross lines with the same width W=1 mm, and the lengths for the horizontal and vertical microstrip lines is 12 mm ($L_{h}$) and 14 mm ($L_{v}$), respectively. The exterior vertical lines have the same width as the two cross lines (1 mm), while the horizontal ones have a width of 0.35 mm.

For tuning purposes of the proposed FSS, a pair of four PIN diodes (MADP-000907-14020) are thought to be used working in the 100 MHz–30 GHz frequency range. Having diodes in the structure requires considering their appropriate placement on the substrates. Hence, as illustrated in Figure 2, cut-slots are inserted for this purpose. Based on the size of the diodes, the width and length of cut-slots are $W_{cut}=0.47$ mm and $L_{cut}=1$ mm. The biasing voltage, which is applied to the structure and controls how the PIN diode operates, also controls how the FSS operates. The behavior of the PIN diode corresponds to a small R (ON-state) when the CN is connected to high voltage, which denotes an almost short-circuit. On the other hand, when low power is provided to the structure, the PIN diodes behave like capacitors (OFF-state) [11]. The diodes realize either a short or a capacitive load, which changes the resonant frequency, so that the structure becomes switchable in function of the voltage drop at the generator terminals.

Two different design structures have been proposed in refs. [11,19]. The first one, which has been designed in a circular shape, has a fundamentally symmetric structure, while the second one, represented by an elliptical structure, has been designed to fulfill requirements such as polarization filtering, polarization conversion, and full notch, which literally needed different symmetries. Moreover, for converting the LP to CP, a $\pm 90^{\circ }$ phase difference between the transmission coefficients for TE and TM incident waves had to be present. The major difference between the structures reported by some of the authors in the previous paper [11] and the design proposed in this work stems from the structure of the CN. Two crossed microstrip lines are used for making connections to the main structure on the surface through via holes.

**Figure 3:**
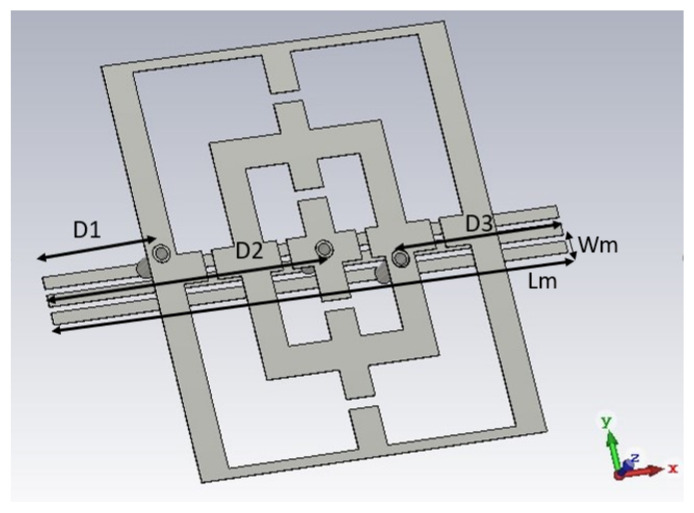
Positions of via-holes in the structure with cut-slots.

In this work, the main FSS structure is connected to three parallel CN microstrip lines by via holes. These lines are located on the opposite side of the PCB with respect to the one with the metallic pattern. Each CN line is linked to one via hole (Figure 3). The dimensions of the microstrip lines placed on the ground face of the structure are $L_{m}$ = 18 mm and $W_{m}$ = 0.4 mm, and the distance between adjacent microstrip lines is 0.6 mm (Figure 3). From an operation point of view, each single microstrip line is biased by a constant potential, giving rise externally to a controllable voltage drop between them. These two values are used to dynamically bias the PIN diodes. In the present design, the lines that provide the same potential to the elements of the FSS are connected together at one of the edges of the PCB. Alternative solutions for local control are possible, but application-dependent.

Furthermore, via-holes are inserted to connect the main structure and feeding lines. The positions of via-holes from the edge of the unit cell in the *x* direction are as follows: the left-hand via-hole is placed at 3.5 mm (D1 in Figure 3), the right via-hole is at 6.5 mm (D3 in Figure 3), and the central via-hole is at 9 mm from the edge (D2 in Figure 3).

**Figure 4:**
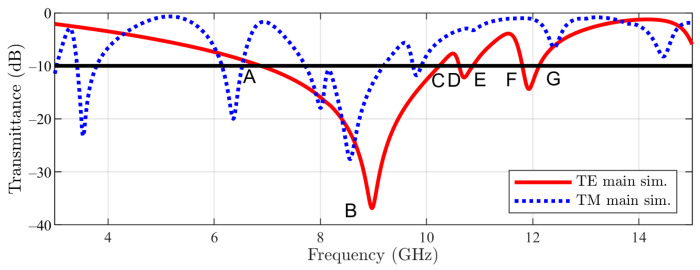
Main unit cell structure result.

For the described geometry of the main structure, the transmission coefficients in normal incidence obtained by means of the simulation tool [20] for both TE- (E field polarized in the *y* direction) and TM-incidence are reported in Figure 4. The normal incidence is defined by ϕ=0 and θ=0 (the angles of spherical coordinates with respect to the reference frame in Figure 1). The frequency domain solver of the cited software has been used, and periodic boundary conditions in the xOy plane have been imposed. In the figure, the following frequency points can be noticed: A, B, C, D, E, F, and G. For TE incidence, a −10 dB stopband between 6.96 GHz (A) and 10.21 GHz (C) with a notch centered at 8.98 GHz (B) can be observed. Furthermore, two smaller bands appear between 10.61 GHz (D) and 10.87 GHz (E) and between 11.80 GHz (F) and 12.12 GHz (G).

**Figure 5:**
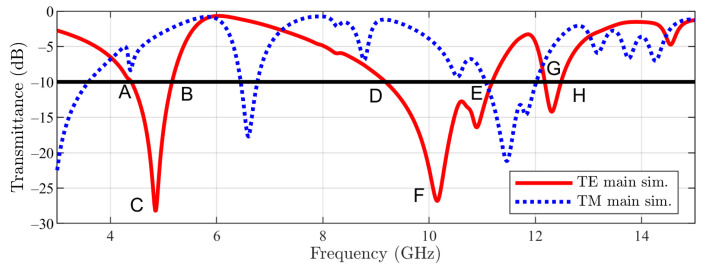
Cut-slot unit cell structure result.

Furthermore, in Figure 5, the cut slot structure simulation results for transmittance are depicted. This time three −10 dB stopbands appear: the first between 4.37 GHz (A) and 5.15 GHz (B) (the notch being centered at 4.86 GHz (C)) and the second between 9.18 GHz (D) and 11.18 GHz (E) with a notch at 10.17 GHz (F). The third one, being the smallest, is between 12.17 GHz (G) and 12.47 GHz (H).

In order to explain the previous results, field images of surface current density are plotted for both main and cut-slot structures.

**Figure 6:**
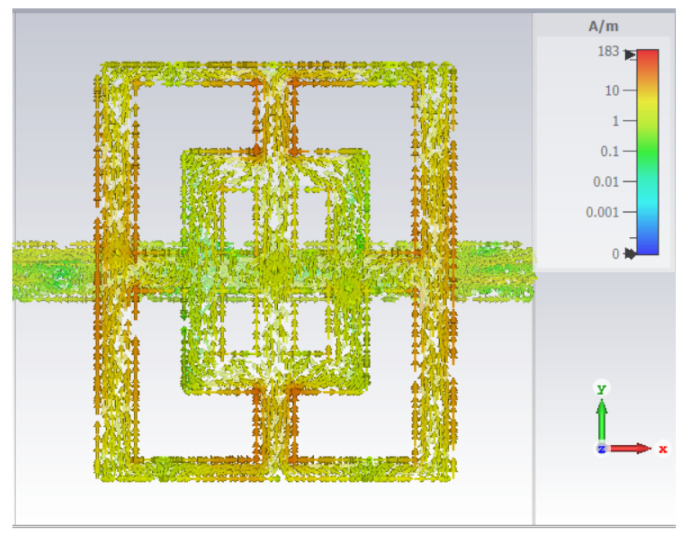
Field images of surface current density, main structure at 8.98 GHz.

Firstly, the main structure is tackled. Given the fact that a resonance centered at 8.98 GHz is present, the selected frequency for field image calculation is 8.98 GHz (Figure 6). In Figure 6, it can be seen that the maximum surface current is 183 A/m for an excitation of 1 V/m. Furthermore, the highest surface currents are visible in the outer part of the structure, thus the large rectangle dimensions ($D_{u}$ and $D_{v}$) represent the origin of the resonance. The current flowing in the orthogonal direction (*x* in this case) is very small, so the cross-pol coupling is also small in this case.

**Figure 7:**
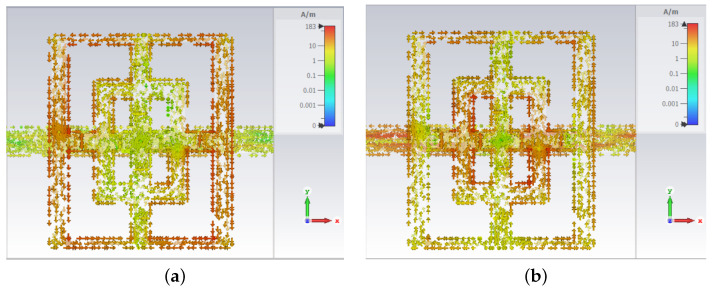
Field images of surface current density, cut-slot structure at 4.86 GHz (**a**), and cut-slot structure at 10.17 GHz (**b**).

Next, the cut-slot structure has two resonances: 4.86 GHz and 10.17 GHz, so the field images are plotted for these two frequencies. The first resonance (4.86 GHz) is inherited from one of the main structures, namely the large rectangle dimensions. This is demonstrated by the high values of surface currents (183 A/m) visible in Figure 7a. Again, the current flowing in the *x* direction has a very small value (Figure 7a).

In the case of the second resonance (10.17 GHz), the small interior rectangle dimensions ($D_{a}$ and $D_{b}$) are responsible. This time the current flowing in the *x* direction is high, with the one in the *y* direction being very small (Figure 7b).

**Figure 8:**
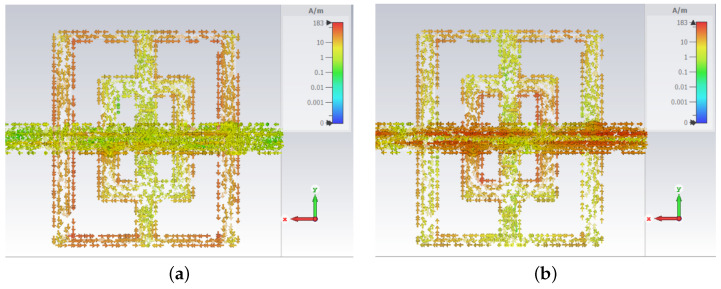
Field images of surface current density, cut-slot structure at 4.86 GHz, CN lines (**a**), and cut-slot structure at 10.17 GHz, CN lines (**b**).

Surface currents have also been evaluated on the opposite side of the structure to assess the influence of the CN lines on the resonances. If Figure 8a is considered, the surface currents at 4.86 GHz have very low values on the CN lines side, while in Figure 8b, high values of currents are present in the CN lines for the second frequency (10.17 GHz). In conclusion, the CN lines have an important but controllable impact on the appearance of the resonance at 10.17 GHz.

**Figure 9:**
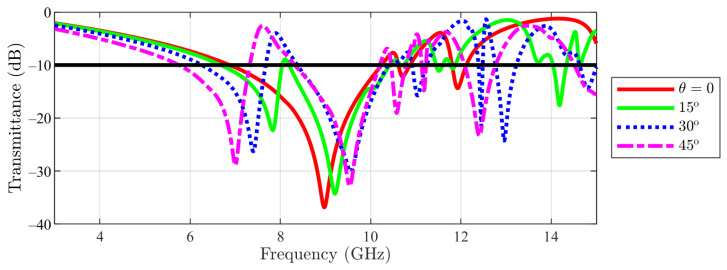
Transmittance coefficient for main structure unit cell at TE incidence field ϕ=0 for different θ values.

Parametric studies on the variation of the transmittance with angle θ for both main and cut-slot structures have been undergone afterwards. Firstly, a parametric study has been carried over for the main structure, in TE incidence. The angle θ has been varied between 0 and 45${}^{\circ }$, in steps of 15${}^{\circ }$. The result from Figure 9 reveals that, by increasing this parameter, the largest notch is shifted to higher frequencies. Furthermore, a second notch appears at lower frequencies.

**Figure 10:**
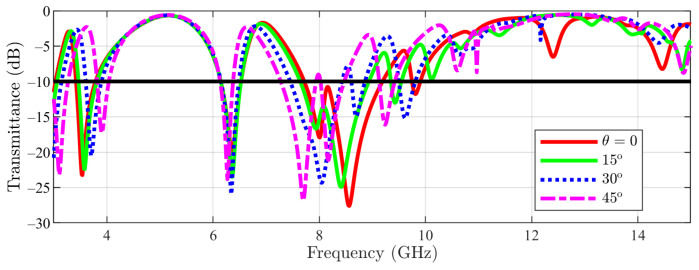
Transmittance coefficient for main structure unit cell at TM incidence field ϕ=0 for different θ values.

Furthermore, in Figure 10 the variation with angle θ of the transmittance of the main structure, in TM incidence is reported. The lower notches (below 4 GHz) are displaced toward higher frequencies, while the notches above 8 GHz are displaced toward lower frequencies.

**Figure 11:**
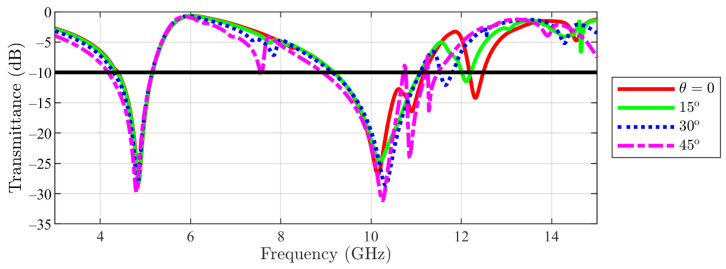
Transmittance coefficient for cut-slot structure unit cell at TE incidence field ϕ=0 for different θ values.

**Figure 12:**
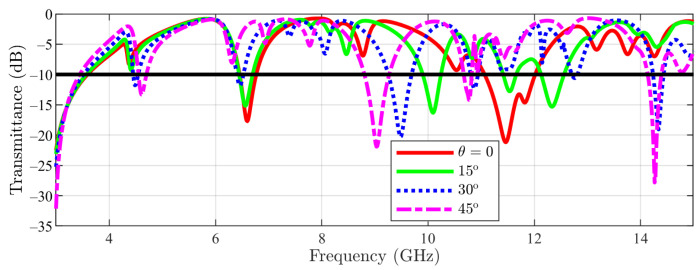
Transmittance coefficient for cut-slot structure unit cell at TM incidence field ϕ=0 for different θ values.

In Figure 11, the result obtained for the transmittance of the FSS for the cut-slot structure in TE incidence is presented. A different behavior than the one for the main structure can be seen. This time, the first notch (≈4.86 GHz) remains almost unchanged, even if θ is varied, while the second stopband slightly expands as θ increases. Then, the result for cut-stlot structure in TM incidence is depicted in Figure 12. Again, the first notch remains constant as θ varies, while the other notches move to lower frequencies.

## 3. Design of Array FSS Structure

Furthermore, to have a better insight into the functionality of the introduced structures in a real environment, when diffraction effects are present, a finite array of unit cells has been simulated. Given the fact that the practical validation will be made on 12 × 12 unit cells on a board, an investigation on the impact of a simulation with more unit cells present was made. Due to simulation resource reasons (time and computer memory usage), simulations were performed with an array consisting of 6 × 6 and 8 × 8 unit cells (for both the main structure and the cut-slot structure).

One simulation with the array having 8 × 8 unit cells lasted up to 80 h, with 6 × 6 unit cells up to 60 h whilst with one unit cell the simulation time was 1 h (on a computer having 192 GB RAM memory and a processor of 2.6 GHz). The calculation was made using the Frequency Domain Solver from CST Studio Suite. A tetrahedral mesh was selected, which results in approximately 72.000 mesh cells. The CPU was accelerated up to two devices, and multithreading was used up to 128 threads. The equation system solver had accuracy of 1 × 10${}^{-3}$.

**Figure 13:**
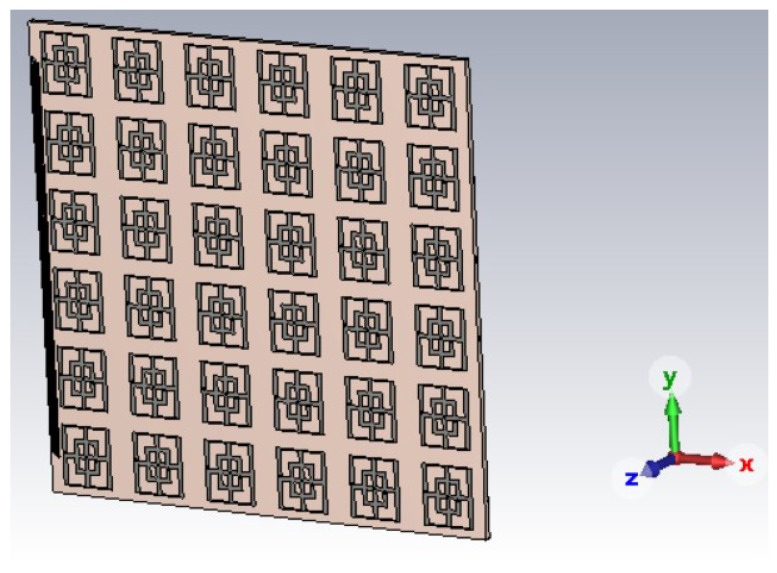
6 × 6 main structure.

The CAD model of the structure with 6 × 6 is visible in Figure 13. For the arrays, the boundary conditions were set at “open” in all three directions (x,y,z), whereas for the unit cell, Periodic Boundary Conditions (PBC) have been used along *x* and *y* and open along the *z* direction.

**Figure 14:**
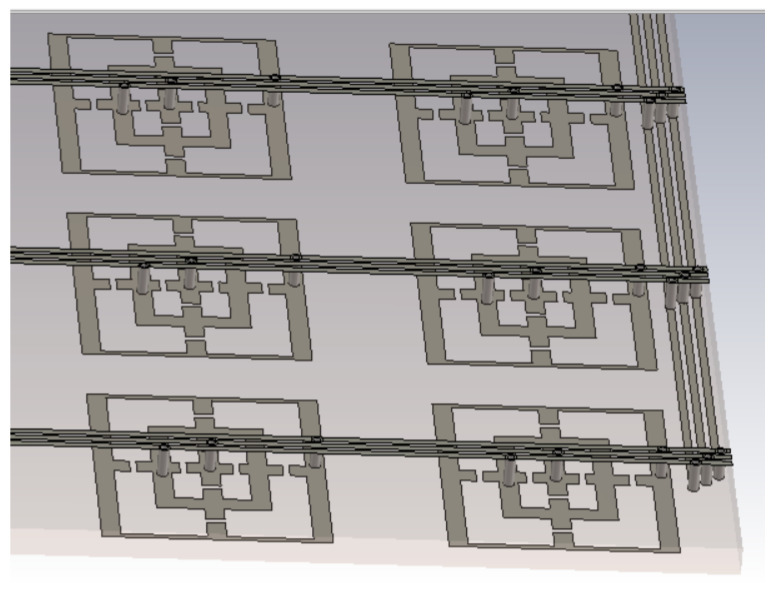
CN lines for array structure.

In Figure 14, the side part of the array structure has been emphasized, where the different biasing lines are joined together. In this figure (for a better rendering, the dielectric has been removed), one can observe the connection of the different microstrip CN lines (here horizontal) for the different rows to the same potential (to the same vertical line). Hence, all rows act in a similar manner.

**Figure 15:**
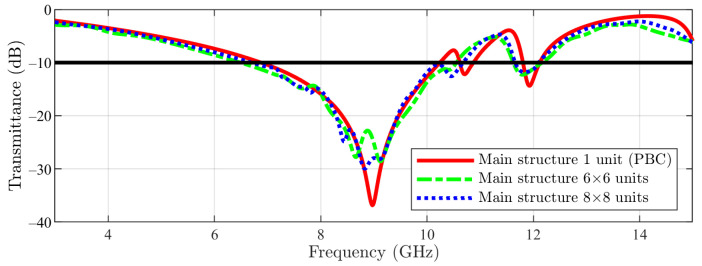
Main structure result: unit cell vs. 6 × 6 array and 8 × 8 array (the −10 dB line is also figured).

In Figure 15, the numerical results for the main structure are visible. For the first stop-band, a similar behavior between 1 unit cell (PBC), 6 × 6 array and 8 × 8 array (with just a slight shift to lower frequencies for the arrays compared with the unit cell) can be noticed. Furthermore, in the case of the arrays, the filtering obtained at the frequency of interest is −22 dB (6 × 6) and −27 dB (8 × 8) in comparison with −36 dB if one unit cell is simulated. Regarding the second stop-band (from higher frequencies), this time a stronger effect is observed: by using the arrays, the stop-band is moved again to lower frequencies, but this time in a stronger manner (≈150 MHz).

**Figure 16:**
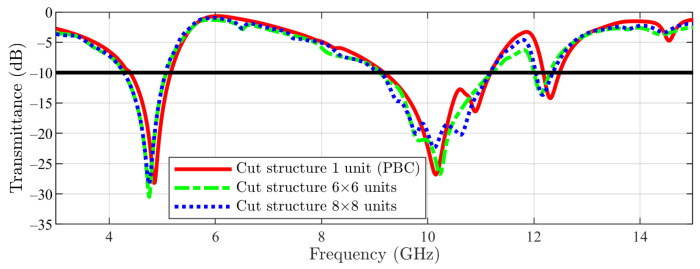
Cut-slot structure result: unit cell vs. 6 × 6 array vs. 8 × 8 array (the −10 dB line is also figured).

The simulation results for the cut-slot structure are depicted in Figure 16. Furthermore, for the first stop-band, a similar behavior between 1 unit cell, 6 × 6 array and 8 × 8 array (with just a slight shift to lower frequencies by the arrays compared with the unit cell side) can be observed. Regarding the second stop-band (from higher frequencies), this time a stronger effect is noticed: by using the 6 × 6 and 8 × 8 arrays, the stop-band is moved again toward lower frequencies, but this time in a stronger manner (≈200 MHz).

**Figure 17:**
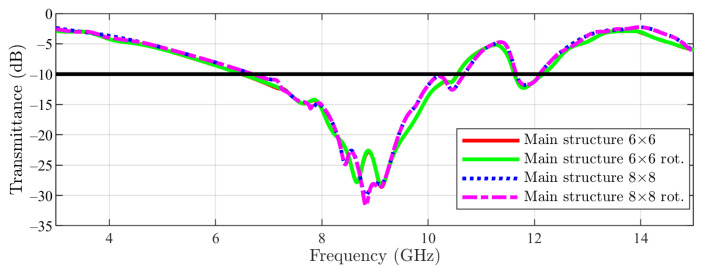
Main structure result (6 × 6 array and 8 × 8 array): E field parallel to CN lines vs. E field perpendicular to CN lines (the −10 dB line is also figured).

**Figure 18:**
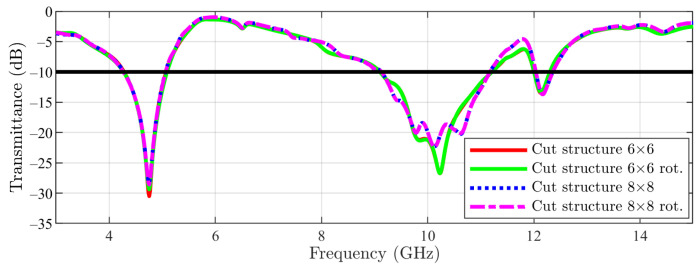
Cut-slot structure result (6 × 6 array and 8 × 8 array): E field parallel to CN lines vs. E field perpendicular to CN lines (the −10 dB line is also figured).

The presented results with arrays were obtained by simulation with linear polarization, with the E field being parallel with the CN lines. Furthermore, the complementary case was studied (E field being perpendicular to the CN lines), with this set of results showing a very good similarity with the previous case (Figure 17 and Figure 18). Main structures with 6 × 6 arrays and 8 × 8 arrays were tackled first (Figure 17), followed by cut-slot structures with 6 × 6 arrays and 8 × 8 arrays in Figure 18.

**Figure 19:**
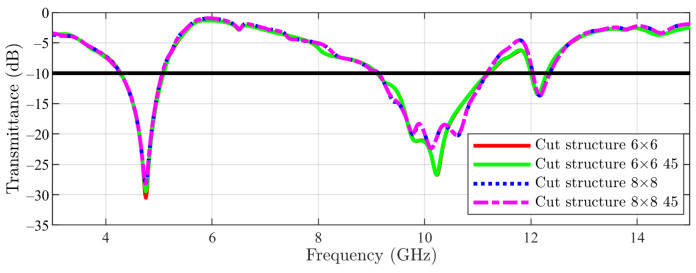
Cut-slot structure result (6 × 6 and 8 × 8 arrays): θ parameter 0 and 45 degrees (the −10 dB line is also figured).

As a final experiment for array structures, an investigation regarding the variation of the transmittance with the angle θ for both main and cut-slot structures has been conducted. The angle was varied between 0${}^{\circ }$ and 45${}^{\circ }$ in steps of 15${}^{\circ }$ for 6 × 6 and 8 × 8 units (Figure 19). Very good consistency is thus obtained in Figure 19, where we can see a comparison between: cut structure 6 × 6 array 0${}^{\circ }$, cut structure 6 × 6 array 45${}^{\circ }$, cut structure 8 × 8 array 0${}^{\circ }$ and cut structure 8 × 8 array 45${}^{\circ }$.

To summarize, the numerical results obtained for the two configurations (6 × 6 and 8 × 8 units) are indistinguishable, hence no further simulations have been carried out. Anyhow, it should be mentioned that the increase in the number of unit cells in the simulations leads to an exponential increase in the unknowns. Considering the similar results between the two reduced dimension configurations, no further simulations on a larger configuration have been performed. The authors would like to underline that, as to the best of their knowledge, such large dimension structures are only marginally reported in the scientific literature.

## 4. Experimental Validation

**Figure 20:**
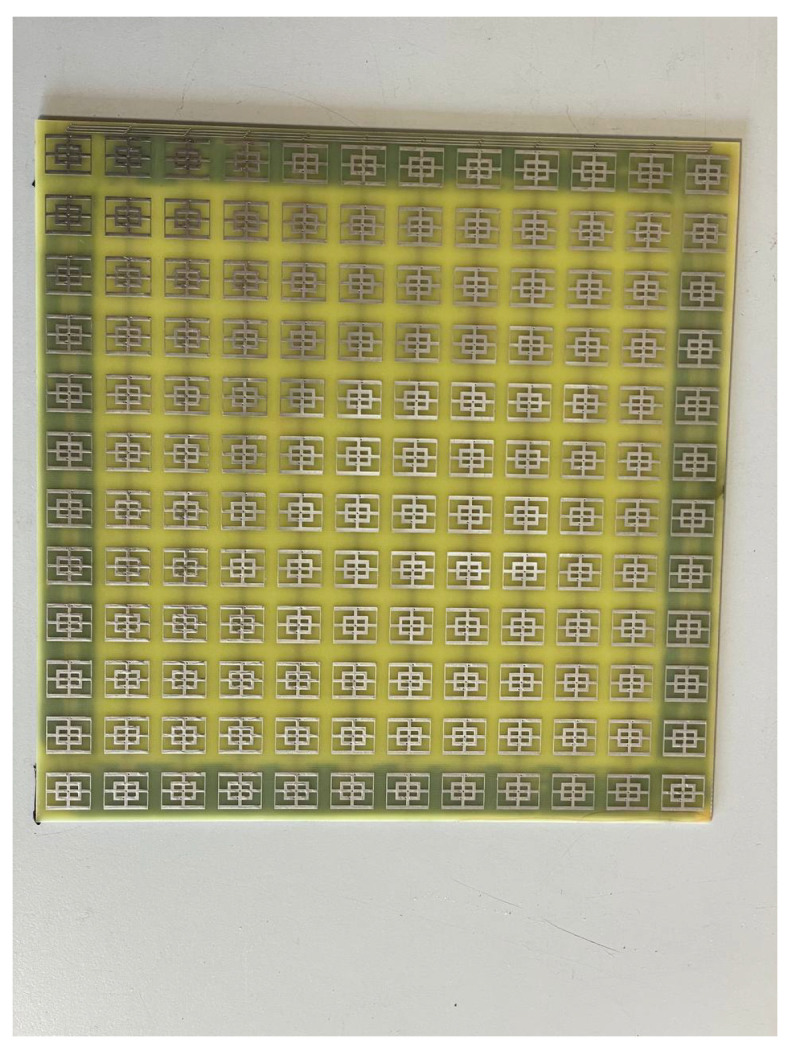
Experimental validation: PCB prototype main structure.

**Figure 21:**
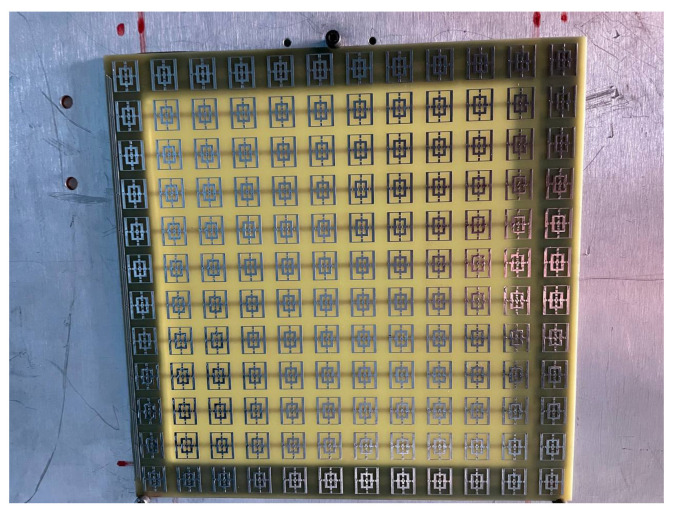
Experimental validation: PCB prototype cut-slot structure.

**Figure 22:**
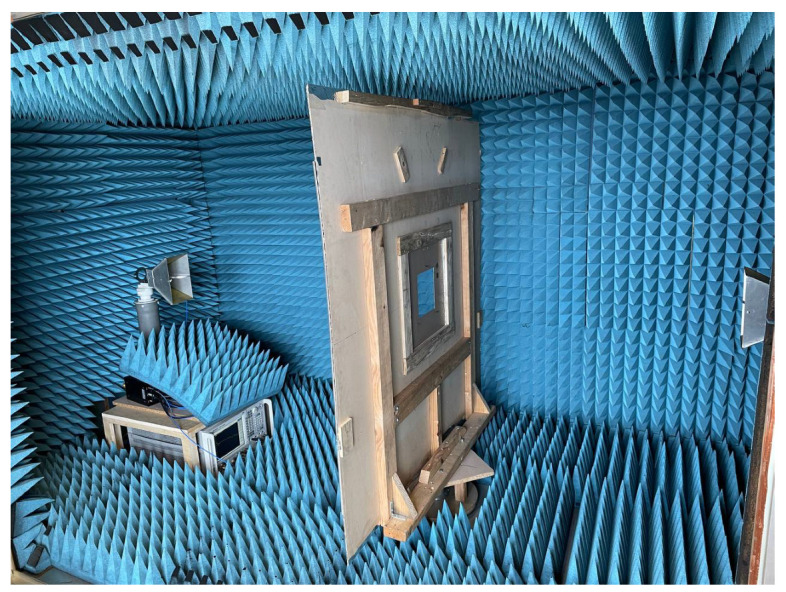
Experimental validation: measurement setup without prototype inserted.

**Figure 23:**
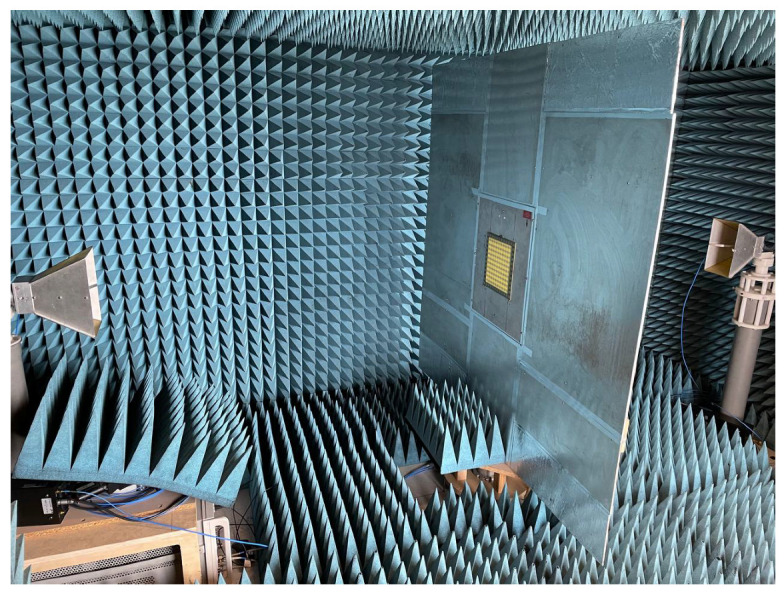
Experimental validation: measurement setup with prototype inserted.

Both of the proposed periodic structures (main and cut-slot) have been realized as prototypes of FR4 printed circuit boards (PCBs) comprising 12 unit cells in each of the two orthogonal directions for a total extension of 216 × 216 mm${}^{2}$. A photograph of Face 1 of the prototype main structure is presented in Figure 20, while Face 1 of the cut-slot structure is visible in Figure 21. Measurements have been performed in an anechoic chamber (Figure 22 and Figure 23) by means of the same substitution method and equipment described in [21].

The FSS’s response varies depending on certain biases. This submitted work only takes into account the most extreme bias levels, where the diodes behave almost like short and open circuits (by using main and cut-slot structures, respectively, we model these scenarios). The practical measurements present intermediary results because, in a future work, the diodes will be placed in the cut-slots to obtain switchability. By using the cut-slots without diodes present, we wanted to verify the feasibility of the cut-slot structure and the effect of switchability on the total stop-band.

**Figure 24:**
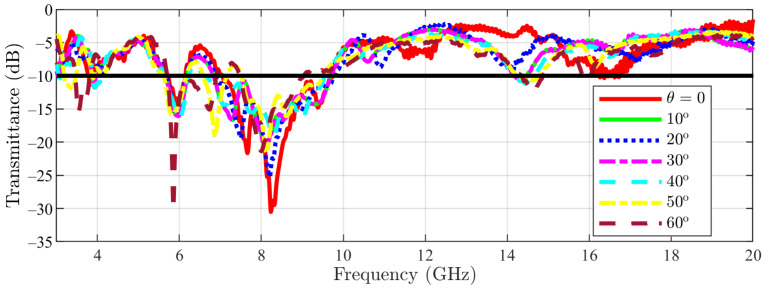
Main structure measurement results (the −10 dB line is also figured).

**Figure 25:**
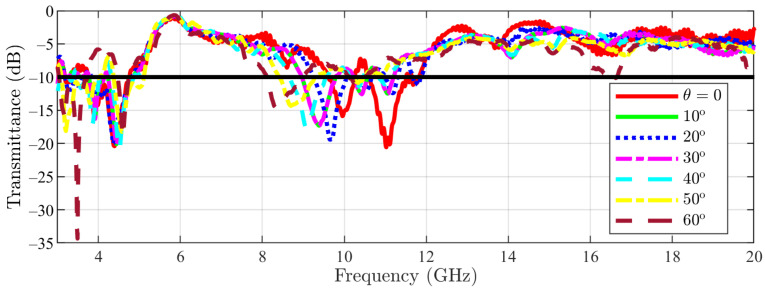
Cut-slot structure measurement results (the −10 dB line is also figured).

To check the stability with respect to the incidence angle, the measured transmittance in normal incidence for the main structure is represented in Figure 24 for colatitudes (angle θ in spherical coordinates) between 0 and 60${}^{\circ }$ in steps of 10${}^{\circ }$. Furthermore, for the cut-slot structure, the same results are visible in Figure 25. This step of 10${}^{\circ }$ step has been chosen similarly to the ones reported in the literature [22,23,24] and also not to burden the graph with too many curves. The authors considered that a 10${}^{\circ }$ is sufficient for assessing the frequency response of the proposed structure in oblique incidence. Moreover, the azimuthal invariance of the frequency response is ensured by the symmetry of the pattern in the unit cell. The effective aperture for the incident waves is reduced by 50% relative to normal incidence, limiting measurements to an angle of incidence of 60${}^{\circ }$ [21].

From the measured data, it appears that an upshift of about 1 GHz of the bandwidth occurs. Furthermore, the bandwidth shrinks when the incidence angle increases. However, there is an overlapping bandwidth of more than 1.6 GHz, i.e., the structure can be used in the 8–9.6 GHz band (corresponding to 1.6/8.8 × 100 = 18.2% relative bandwidth).

**Figure 26:**
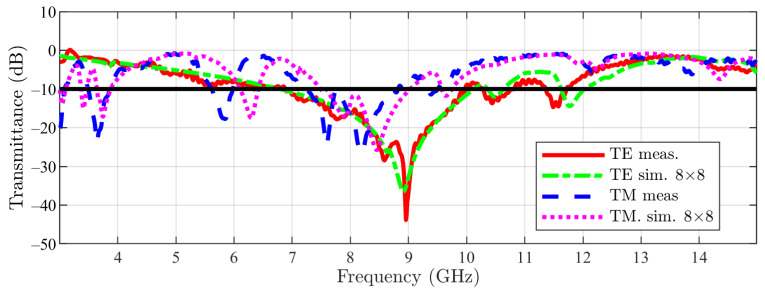
Main structure measurement vs. simulation results (the −10 dB line is also figured).

**Figure 27:**
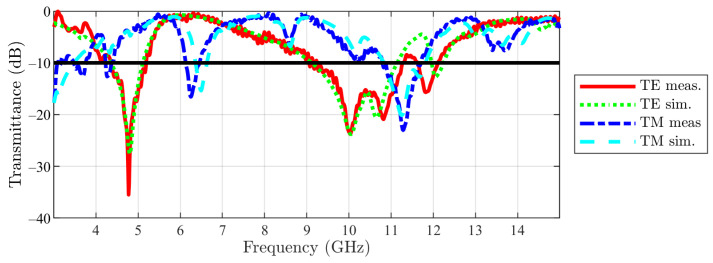
Cut-slot structure measurement vs. simulation results (the −10 dB line is also figured).

Next, the simulation results have been compared with the measurement ones. The measured transmittances for the main structure in both incidences (TE-red and TM-dark blue dotted) are represented in Figure 26, together with the simulated ones (TE-green dotted and TM-light blue dotted), in order to reveal the good matching between the two. Respectively, for the cut structure, the comparison is visible in Figure 27, with the color code remaining the same. The reported data show a good agreement between simulation and measurement results. For low values of the transmittance, i.e., in the stop-band, there are some negligible variations that are brought on by differences in the configuration and irregularities in the PCB’s dielectric, by tolerances in the metalization, and by higher-order modes that are launched as surface waves and can radiate when they reach the PCB’s boundaries [21].

## 5. Conclusions

In this paper, a switchable FSS with different configurations, operating as a band-stop spatial filter for frequencies below 12 GHz, has been proposed. Two structures (main and cut-slot), having incorporated a CN, show filtering in distinct frequency bands. The structures have been implemented on a cost-effective FR4 substrate. By undergoing a parametric study for the colatitude angle, the switchability of the proposed structures has been demonstrated.

Furthermore, a FSS array has been designed with 6 × 6, and 8 × 8 unit cells in order to compare more accurately with the practical validation. To the best of the author’s knowledge, such an approach has little consideration in the existing literature. The structures have been assessed by simulation and measurement in an anechoic room. The obtained results demonstrate a good agreement between theory and experiments.

The advantage of the proposed solution stems from the interplay between the biasing network and the structure of the metal pattern of the unit cell. Namely, the biasing network consists of microstrip lines, so that its impact can be assessed as part of the electromagnetic design. This is not the case for other solutions, e.g., when using wires to bring bias to the switching of tuning elements. The switchability of the proposed structure is achieved by using the main and cut-slot structures. They model the short and open circuits of PIN diodes, whose bias determines the values of the elements in the equivalent circuit. At different biases, the response of the FSS changes accordingly.

The authors consider that the reported change in the band-stop structure of the FSS is quite significant. In the TE incidence case, three stop-bands in the intervals (6.96, 10.21) GHz, (10.61, 10.87) GHz, and (11.80, 12.12) GHz for the main structure, which commute to (4.37, 5.15) GHz, (9.18, 11.18) GHz, and (12.17, 12.47) GHz for the cut-slot one, have been obtained. Note the switching between narrow- and wideband behavior of the second stop-band. Similar dynamics of the stop-bands can be noticed for a TM incidence.

## Data Availability

Not applicable.
